# Purification, characterization, immobilization and applications of an enzybiotic β-1,3–1,4-glucanase produced from halotolerant marine *Halomonas meridiana* ES021

**DOI:** 10.1007/s11274-023-03527-1

**Published:** 2023-02-06

**Authors:** Eman E. Gadallah, Aliaa M. El-Borai, Samy A. El-Aassar, Ehab A. Beltagy

**Affiliations:** 1 Botany and Microbiology Department, Faculty of Science, Alexandria University, Alexandria, Egypt; 2 National Institute of Oceanography and Fisheries (NIOF), Cairo, Egypt

**Keywords:** Anti-bacteria, Anti-fungi, DEAE anion exchanger, Enzybiotic, Glucanase

## Abstract

**Abstract:**

Extracellular β-1,3–1,4-glucanase-producing strain *Halomonas meridiana* ES021 was isolated from Gabal El-Zeit off shore, Red Sea, Egypt. The Extracellular enzyme was partially purified by precipitation with 75% acetone followed by anion exchange chromatography on DEAE-cellulose, where a single protein band was determined with molecular mass of approximately 72 kDa. The K_m_ value was 0.62 mg β-1,3–1,4-glucan/mL and V_max_ value was 7936 U/mg protein. The maximum activity for the purified enzyme was observed at 40 °C, pH 5.0, and after 10 min of the reaction. β-1,3–1,4-glucanase showed strong antibacterial effect against *Bacillus subtilis*, *Streptococcus agalactiae* and *Vibrio damsela*. It also showed antifungal effect against *Penicillium* sp. followed by *Aspergillus niger*. No toxicity was observed when tested on *Artemia salina*. Semi-purified β-1,3–1,4-glucanase was noticed to be effective in clarification of different juices at different pH values and different time intervals. The maximum clarification yields were 51.61% and 66.67% on mango juice at 40 °C and pH 5.3 for 2 and 4 h, respectively. To our knowledge, this is the first report of β-1,3–1,4-glucanase enzyme from halotolerant *Halomonas* species.

**Graphic Abstract:**

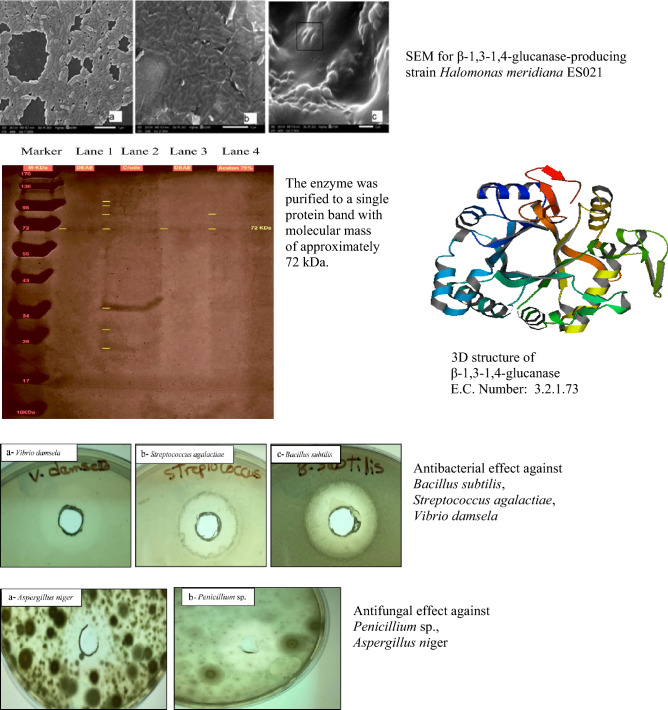

## Introduction

β-1,3–1,4-glucanase (Ec.3.2.1.73, lichenase) is an industrially important enzyme because of its strict specificity for β-glucan cleavage (Jang et al. 2021). β-1,3–1,4-glucanase is a glycoside hydrolase involved in β-1,3–1,4-glucans enzymatic depolymerization (Zalila-Kolsi et al. 2018). It exhibits a strict substrate specificity for cleavage of β-1,4 glycosidic bonds (Sun et al. 2012). It precisely cleaves the β-1 → 4 glycosidic linkage of 3-O-substituted glucose in 1,3–1,4-β-D-glucan, resulting in β-glucan oligosaccharides with a degree of polymerization of three or four, with a connected bonds made up of two or three β-1 → 4 linkages separated by one β-1 → 3 linkage, similar to barley β-glucan structure (Ghotra et al. 2008). Thus, when β-glucan is enzymatically degraded by β-1,3–1,4-glucanase, β-glucan oligosaccharides with a degree of polymerization of three or four can be formed (Cho et al. 2018).

Using immobilized enzymes in chemical, pharmaceutical and food industries has become a routine process with high substrate specificity, high catalytic activity, and mild optimal reaction conditions (Basso and Serban 2019; Cao et al. 2016). The most significant advantages of enzyme immobilization are reusability and the simplicity with which it can be separated (Cho et al. 2018). Furthermore, the remaining enzyme amount left in the product is reduced, and enzyme immobilization allows enzymes to be reused multiple times, lowering enzyme consumption costs (Sheldon 2007). Furthermore, enzyme immobilization frequently results in increased heat stability or resistance to mechanical inactivation (Tu et al. 2006).

Enzybiotics are a new class of anti-microbials based on enzymes that may present a solution to this global demand. The term enzybiotics is a hybrid from the two words enzyme and antibiotic (Wu et al. 2012). Enzybiotics are a solution for antibiotic resistance problem using natural antimicrobial enzymes or entire bacteriophages to inhibit pathogenic bacteria or fungi growth (Veiga-Crespo and Villa 2009). A study by Jin et al. (2011) showed that β-1,3–1,4-glucanase from endophytic *Bacillus subtilis* could be a desirable vital agent against microbial pathogens with higher efficiency and lower toxicity, they proved that the bacterial enzyme had a broad antimicrobial spectrum against fungal and bacterial strains. Antifungal effect of β-1,3–1,4-glucanase was also reported by Dewi et al. (2016), Xu et al. (2016), Zalila-Kolsi et al. (2018) and Yuan et al. (2020).

Fruit juices are cloudy in different degrees because they contain polysaccharides (pectin, lignin, cellulose, hemicelluloses and starch), proteins and some metals (Vaillant et al. 2001). So, enzymes play a key role in the production of fruit. Their main goals are increasing juice extraction from raw materials, producing a clear and visually appealing finished product and improving processing efficiency as solid settling, pressing, or removal (Sharma et al. 2014).

In this report, purification, immobilization and characterization of the extracellular β-1,3–1,4-glucanase from the halotolerant marine isolate *Halomonas meridiana* will be described, seeking for potent features and proposing for multiple applications with highly efficient mode of action. Moreover, we aimed to scan its enzybiotic activity against a broad spectrum of microbial strains.

## Materials and methods

### Extracellular β-1,3–1,4-glucanase production

The bacterial isolate producing β-1,3–1,4-glucanase was isolated from Gabal El-Zeit-off shore, Red Sea, Egypt and identified as *Halomonas meridiana* ES021 by partially sequencing of 16S rRNA gene that was previously reported by the same authors (El-Borai et al. 2022). Zobell medium was used for maintenance of the bacterial strain throughout the work which contained the following ingredients (g/L): yeast extract, 1.0; peptone, 5.0; FeSO_4_.7H_2_O, traces; agar–agar, 15; filtered sea water 800 mL; distilled water 200 mL and pH was adjusted to 7.5 (Zobell 1942; Koedooder et al. 2018). While semisynthetic medium containing barley coupled with wheat flour, which were rich with the polysaccharide β-1,3–1,4-glucan as the major water-soluble constituent (Ghotra et al. 2008; Skendi et al. 2003), was used for enzyme production, contained the following ingredients (g/L): barley flour, 135; wheat flour, 44.8; urea, 4.0; fructose, 5.0; KH_2_PO_4_, 0.625; MgSO_4_.7H_2_O, 0.1 and CaCl_2_, 0.1; inoculum size, 3%. The pH was adjusted to 8.0 and the incubation was carried out statically at 37 °C for 16 h.

### Extraction of β-1,3–1,4-glucan from barley flour

The substrate used for enzyme activity assay was β-1,3–1,4-glucan polysaccharide extracted in laboratory from whole barley flour according to the procedure described by Wood et al. (1977).

### Purification of extracellular β-1,3–1,4-glucanase produced by *Halomonas meridiana* ES021

The culture broth was centrifuged at 5000 rpm for 10 min in a cooling centrifuge at 4 °C. 75% acetone concentration was used to precipitate the protein content of supernatant of centrifuged *Halomonas meridiana* ES021 cultures. The fraction was dialyzed against distilled water (Niu et al. 2016b). The resulting protein fraction was introduced to DEAE-Cellulose A-52 column (28 × 1.8 cm), which was equilibrated with 0.05 M tris–HCl buffer pH 8.0. The gradual elution of the protein was done using 0.05 M tris–HCl buffer pH 8.0 followed by different molarities (0.1, 0.2, 0.4, 0.6, 0.8 and 1.0 M) of NaCl dissolved in the same buffer. The eluent was obtained in 3.0 mL fractions collected at a flow rate of 1.0 mL/min adjusted with a peristaltic pump.

### Protein electrophoresis

According to the described protocol steps of Laemmli (1970), Purified protein was detected after running on SDS-PAGE (8%) (Huang et al. 2022).

### Enzyme assay and protein determination

Following the protocol of Miller (1959), β-1,3–1,4-glucanase activity was measured depending on the reducing sugars released as a result of barley β-1,3–1,4-glucan hydrolysis (Huang et al. 2022) where one activity unit of β-1,3–1,4-glucanase activity (U) was defined as the amount of enzyme required to produce 1.0 µmol reducing sugar (glucose equivalent) per minute from β-1,3–1,4-glucan. As described by Lowry et al. (1951), the enzyme protein content was also measured (Ma et al. 2021).

### Characterization of purified extracellular β-1,3–1,4-glucanase

To determine the optimal substrate concentration, different substrate concentrations varying from 0.1 to 3.0 mg/mL were added to 100 µL equivalent to 12 µg of purified enzyme solution. Lineweaver and Burk (1934) method was applied for kinetic parameters determination providing the Michaelis–Menten equation: V_1_ = V_max_ [S] / K_m_ + [S], where the Michaelis–Menten constant (K_m_) and maximal velocity (V_max_) were calculated upon the equation symbols V_1_, [S], K_m_ and V_max_ that were identified as the reaction velocity, the substrate concentration, the substrate concentration at half-maximal velocity, and the maximal velocity, respectively. To determine the optimal temperature for the β-1,3–1,4-glucanase activity, enzyme reactions were carried out at different temperatures ranging from 30 to 70 °C for 10 min. The pH dependence of β-1,3–1,4-glucanase activity was determined, the range of studied pHs were from 3.0 to 8.0 (Lim et al. 2022**)**.

Thermal stability of the enzyme was evaluated by preheating certain portions of the enzyme preparation without substrate separately at different temperatures (40, 50, 60 and 70 °C), for various time periods (15, 30 and 60 min), respectively. The optimal incubation period for β-1,3–1,4-glucanase activity was determined by carrying out the reaction at different time intervals ranging from 5 till 80 min. Without adding the substrate, different NaCl concentrations ranging from 10 to 90 ppt were exposed to the enzyme to evaluate the effect of salinity.

To assay the effect of some activators and inhibitors on β-1,3–1,4-glucanase activity, the purified enzyme solution was preincubated for 1 h at room temperature (25 °C) with the different tested substances (Ni^++^, Co^++^, Cu^++^, Cr^++^, Fe^++^, Mn^++^, Mg^++^, Ca^++^, SDS, acetic acid and urea) each at a time, at concentrations 0.005 M and 0.05 M.

### Immobilization of semi-purified β-1,3–1,4-glucanase enzyme produced from *Halomonas meridiana* ES021 cultures

#### Immobilization of semi-purified β-1,3–1,4-glucanase by physical adsorption

This protocol was carried out according to Woodward (1985). Chitin, Chitosan and silica gel were used as solid supports to immobilize β-1,3–1,4-glucanase enzyme by adsorption. 0.1 g of each solid support was incubated with 1.0 mL semi-purified β-1,3–1,4-glucanase enzyme solution dissolved in 2.0 mL of 0.0.5 M phosphate buffer pH 6.0 at ambient temperature overnight (Beltagy et al. 2022).

#### Immobilization of semi-purified β-1,3–1,4-glucanase by ionic bonding

This method was carried out according to Woodward (1985), where anion exchanger (DEAE-cellulose) equilibrated with 0.05 M tris–HCl buffer pH 9.0 and cation exchanger (CM-cellulose) equilibrated with 0.05 M phosphate buffer pH 6.0 were incubated with 1.0 mL semi-purified β-1,3–1,4-glucanase enzyme solution (1425.60 U/mg protein) at ambient temperature overnight (Beltagy et al. 2022).

#### Immobilization of semi-purified β-1,3–1,4-glucanase by entrapment

0.1 g agar or agarose was dissolved in 10 mL distilled water to form 1% agar or agarose gel. 2.0 mL of each gel material were mixed with 1.0 mL of semi-purified β-1,3–1,4-glucanase solution, then poured into a Petri-dish. After gel solidification, equal cubes cut with 0.5 cm diameter using sterile cutter were applied to the reaction after washing several times to get rid of any unbound enzyme. Ca-alginate gel was prepared by stirring 0.1 g Na-alginate in 10 mL distilled water to form 1% Na-alginate gel. Kappa-carrageenan gel was prepared by dissolving 0.1 g kappa-carrageenan in 10 mL distilled water to form 1% kappa-carrageenan gel. The entrapment was structured by adding the mixture composed of 2.0 mL of each gel material mixed with 1.0 mL of semi-purified β-1,3–1,4-glucanase enzyme solution, through a syringe into 50 mL of 2% calcium chloride solution (for Ca-alginate) or 2% potassium chloride solution (for kappa-carrageenan) and left for 2 h. The beads formation of 1.0–1.5 mm diameter were collected and used in the reaction after washing several times to remove the unbound enzyme.

#### Immobilization of semi-purified β-1,3–1,4-glucanase by covalent bonding

The gel was prepared by dissolving 0.5 g chitosan in 15 mL acetic acid (2.5%) then dropped through a syringe into 100 mL sodium hydroxide solution (1.5%) to form beads and left for 1 h. The resulting beads were divided into 3 portions (1.0 g each), added to different concentrations of glutaraldehyde solution (1, 3 and 5%) and left overnight at ambient temperature. After that, the beads were rinsed with distilled water, 0.5 g of each portion was mixed with 1.0 mL semi-purified β-1,3–1,4-glucanase enzyme solution and left at ambient temperature overnight. After equilibration, glutaraldehyde was decanted and beads were used in reaction after washing several times.

#### Assay of immobilized enzyme

The immobilized enzyme was incubated with 1.0 mL of 0.2% barley β-glucan dissolved in 0.1 M phosphate buffer pH 6.0 for 10 min. After the desired time of incubation, the reaction solution was separated from the immobilized enzyme; the enzyme activities of unbound and immobilized enzyme were assayed using DNS method described by Miller (1959).

#### Graphing and statistical analysis

Excel software was used for graphing and expressing results as mean ± SD.

### Applications of extracellular β-1,3–1,4-glucanase enzyme produced by *Halomonas meridiana* ES021 cultures

#### Enzybiotic activity of purified β-1,3–1,4-glucanase

Antimicrobial susceptibility testing was performed with agar well diffusion method. Nutrient agar plates for bacterial pathogens, and potato dextrose agar plates for fungal pathogens were inoculated by the microbial pathogens using pour plate technique. 25 µg (200 µL) of enzyme protein solution was introduced into the well and allowed to diffuse in agar media before incubation (Jin et al. 2011). The bioassay plates were incubated overnight at 37 °C for bacteria and for 24 and 48 h at 30 °C for fungi to measure the diameter of the inhibition zone (mm) to evaluate antimicrobial effect.

#### Juice clarification by semi-purified β-1,3–1,4-glucanase

Juice clarification was carried out by adding 25 mg enzyme protein to 5.0 mL of each fruit juice in different sets and control was done (1.0 mL of distilled water was added to each of the fruit juice). Reaction mixtures were incubated for 2 and 4 h at 40 °C. Transmittance of sample is determined at 650 nm. Juice clarification in percent (%) was calculated by formula: (%) Clarification = (T_t_-T_c_/T_c_) × 100, where, T_t_ is transmittance of test and T_c_ is transmittance of control (Kothari et al. 2013).

#### Toxicity of purified β-1,3–1,4-glucanase

The test was performed using *Artemia salina* larvae (Obtained from NIOF, Alexandria, Egypt). An artificial saline solution was prepared using sea water and distilled water in ratio 1:2, 10 of *Artemia salina* larvae were added to enzyme protein solutions at different concentrations (20–120 µg/mL) prepared by diluting the extract in artificial saline solution. The larvae were incubated with the saline solution in negative control (without added enzyme) (Meyer et al. 1982; Cavalcante et al. 2019). The assay was maintained under artificial lighting with aeration at 27 ± 2 °C and mortality rates were determined. After 24 and 48 h the number of dead and live larvae in each vial was counted and the probability of mortality was calculated according to the formula: Mortality probability (%) = (r/n) × 100, where, r is number of dead larvae and n is total number of *Artemia salina* in each vial.

## Experimental results

### Anion exchange chromatography on DEAE-cellulose

The crude enzyme was first partially purified by fractional precipitation with 75% acetone fraction, which yielded the highest specific activity (1425.60 U/mg protein) hitting 2.39-fold higher than that obtained from the crude enzyme (data not shown).

**Fig. 1:**
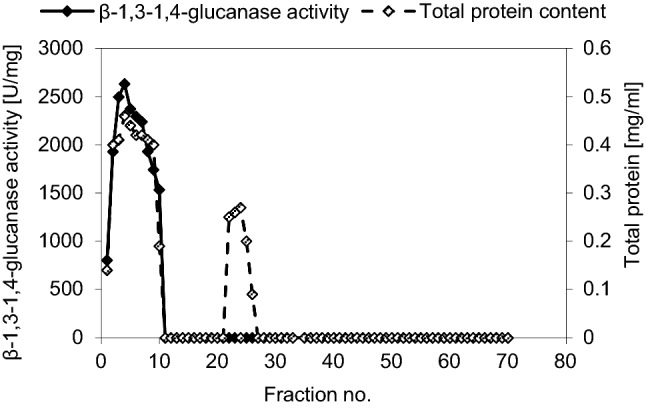
Purification of the semi-purified β-1,3–1,4-glucanase enzyme using on exchange chromatography on DEAE Cellulose A-52; showed the first protein peak which accompanied with the only activity peak recovered from the column, the second protein peak and no enzyme activity noticed with this peak

A certain weight 8.12 mg protein of the partially purified enzyme was dissolved in 0.05 M tris–HCl buffer pH 8.0 and was loaded on DEAE-cellulose A-52 column that was equilibrated with the same buffer. The eluted fractions were 70 fractions as shown in Fig. 1, where about 58.6% of the applied enzyme protein was recovered by the eluting solutions and was separated to two protein components. The first protein component was the major one; it was covered by fractions from 1 to 10 representing about 77.52% of the total recovered protein. The second protein peak was a minor one and was covered by fractions from 22 to 26 representing about 22.48% of the total recovered protein. The total recovered β-1,3–1,4-glucanase activity from the column was fractionated in the column into 1 peak synchronized with the first protein peak, where it represented about 65.28% of the original activity. 3.4 mg of the purified enzyme was obtained with specific activity reached about 2409 ± 15.54 U/mg and a purification of 3.87-fold of the crude enzyme and a recovery yield about 5.96 ± 0.12%. A summary of the purification steps of β-1,3–1,4-glucanase enzyme is shown in Table 1.

**Table 1:** Summary of purification steps of β-1,3–1,4-glucanase enzyme produced from *Halomonas meridiana* ES021

Purification step	Total protein (mg)	Total activity (U)	β-1,3–1,4-glucanase activity (U/mg)	Purification fold	Yield (%)
Crude enzyme	223 ± 7.00	137,375 ± 5494	616 ± 5.31	1	100
Acetone precipitation (75%)	8.16 ± 0.25	12,022 ± 420	1473 ± 6.34	2.39 ± 0.01	8.75 ± 0.04
Ion-exchange chromatography	3.4 ± 0.09	8190 ± 164	2409 ± 15.54	3.87 ± 0.03	5.96 ± 0.12

### Gel electrophoresis

**Fig. 2:**
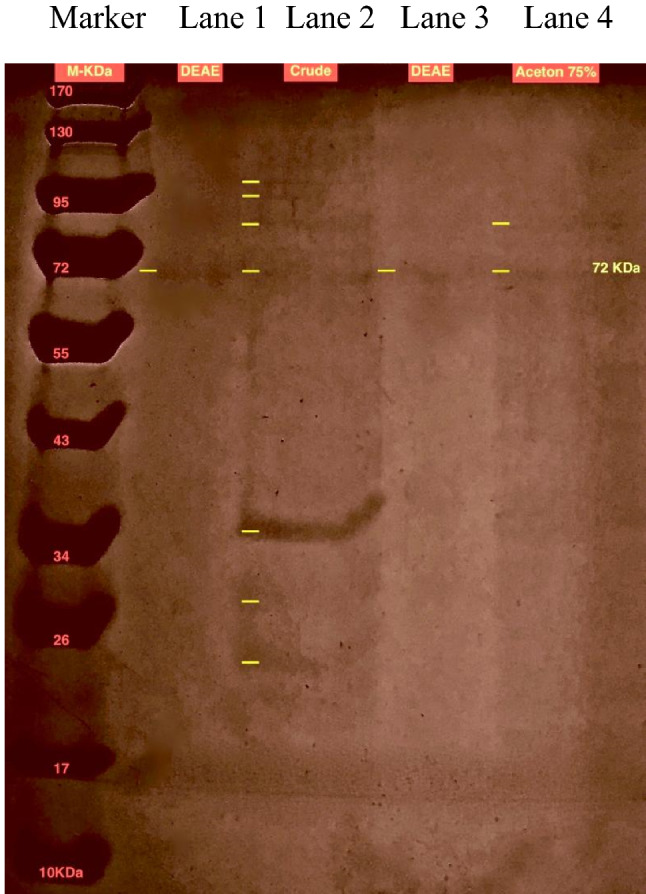
SDS-PAGE of samples during purification of β-1,3–1,4-glucanase from *Halomonas meridiana* ES021. The standard protein marker (Page Ruler prestained protein ladder (10–170 kDa). Lane 1&3: replica for the fraction eluted from DEAE-Cellulose A-52 column (pure enzyme); lane 2: culture supernatant (crude enzyme); lane 4: 75% acetone fraction (semi-purified enzyme)

The purity, integrity and molecular weight of the β-1,3–1,4-glucanase enzyme was examined by gel electrophoresis. β-1,3–1,4-glucanase enzyme obtained from the ion exchange column gave a single band on SDS-PAGE gel, indicating the purity and integrity of the isolated β-1,3–1,4-glucanase, and the molecular weight of the purified enzyme was estimated to be 72 kDa (Fig. 2).

### Characterization of purified β-1,3–1,4-glucanase enzyme

#### Optimum conditions

**Fig. 3:**
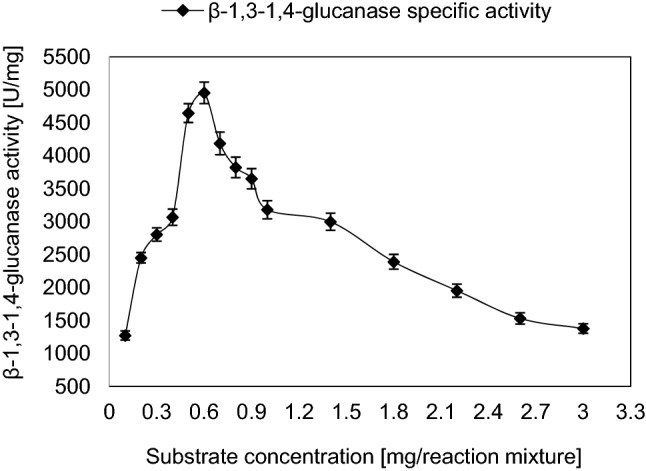
Effect of substrate concentration on the activity of the β-1,3–1,4-glucanase from *Halomonas meridiana* ES021. Enzyme activity increased gradually by increasing substrate concentration, the maximal enzyme activity was reached at substrate concentration of 0.6 mg/reaction mixture, further increase in substrate concentration yielded lower enzyme activity

**Fig. 4:**
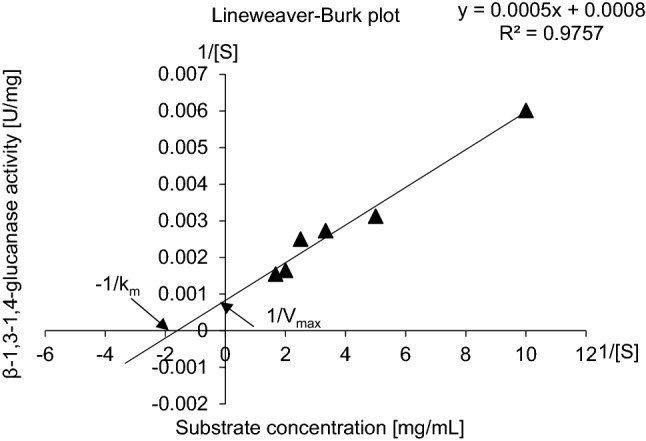
Lineweaver–Burk plot for evaluation of kinetic constants (K_m_ and V_max_) β-1,3–1,4-glucanase from *Halomonas meridiana* ES021. Enzyme activity was determined at different β-1,3–1,4-glucan concentrations of 0.1–3.0 mg/ml. V_max_ value of the enzyme was estimated to be 1111 U/mL (7936 U/mg protein) when the K_m_ is 0.62 mg β-1,3–1,4-glucan/mL

The effect of substrate concentration on the activity of the purified β-1,3–1,4-glucanase enzyme illustrated the correlation between the rate of the reaction and the substrate concentration. The optimum substrate concentration for the pure enzyme was 0.6 mg/reaction mixture giving β-1,3–1,4-glucanase specific activity about 4954 ± 162 U/mg which was about 2.07-fold of that obtained by initial substrate concentration (Fig. 3). The K_m_ and V_max_ values of the purified enzyme were found to be 0.62 mg β-1,3–1,4-glucan/mL and 1111 U/mL equivalent to specific activity 7936 U/mg protein, respectively (Fig. 4).

**Fig. 5:**
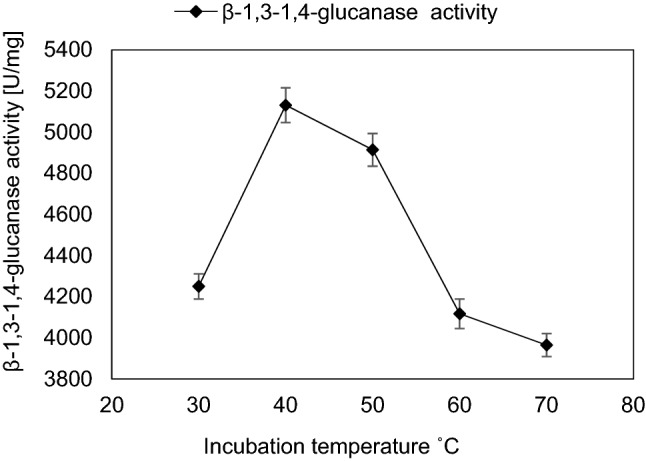
Effect of incubation temperature on the activity of purified β-1,3–1,4-glucanase from *Halomonas meridiana* ES021, the enzymatic reaction was carried out at temperature range from 30 to 70 ℃, the enzyme retained more than 80% of its activity in a temperature within this range, and the maximal enzyme activity was reached at 40 ℃

**Fig. 6:**
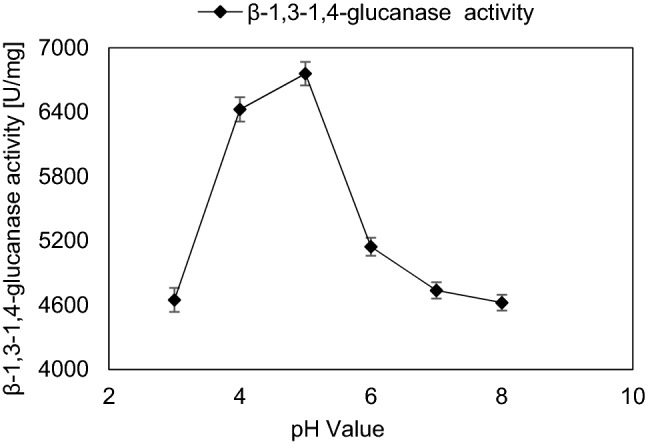
Effect of pH value on the activity of the purified β-1,3–1,4-glucanase enzyme from *Halomonas meridiana* ES021, different pH values ranging from 3.0 to 8.0 were tested using four buffers, 0.05 M glycine HCl buffer, 0.05 M acetate buffer, 0.05 M phosphate buffer and 0.05 M tris–HCl buffer, the maximal enzyme activity was reached at pH 5.0, the enzyme retained more than 90% of its activity in a pH range from 4.0 to 7.0

**Fig. 7:**
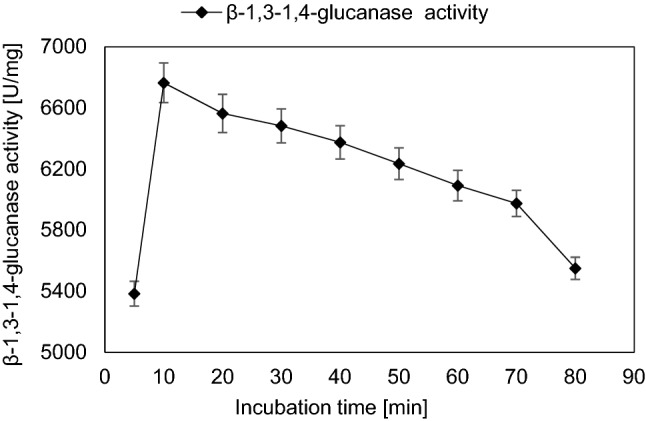
Effect of incubation time on the activity of purified β-1,3–1,4-glucanase from *Halomonas meridiana* ES021, the enzymatic reaction was carried out at 40 ℃ and pH 5.0 for time intervals ranging from 5 to 80 min, and the maximal enzyme activity was achieved after 10 min of incubation, further increase in incubation time causes gradual slight decrease in enzyme activity

The influence of temperature, pH and incubation time of the reaction using a substrate concentration of 0.62 mg/mL reaction mixture was studied. The optimum temperature was 40 °C with maximum β-1,3–1,4-glucanase activity which was 5131 ± 147 U/mg which was 1.04-fold of that obtained by initial incubation temperature (Fig. 5). At 70 °C, the enzyme activity was 77.28% of that obtained at 40 °C. The optimum pH value was pH 5.0 with β-1,3–1,4-glucanase activity equals to 6759 ± 109 U/mg which was about 1.3-fold increase than that obtained by initial pH value (Fig. 6). β-1,3–1,4-glucanase enzyme showed to be a relatively stable at pH range from 4.0 to 6.0. The lowest enzyme activity was observed at pH 8.0 showing about 31.59% decrease of value obtained at pH 5.0. The optimum incubation time of reaction mixture was 10 min with β-1,3–1,4-glucanase activity about 6765 ± 130 U/mg (Fig. 7). Further increase in incubation period causes gradual slight decrease in enzyme activity.

#### Thermal stability

**Fig. 8:**
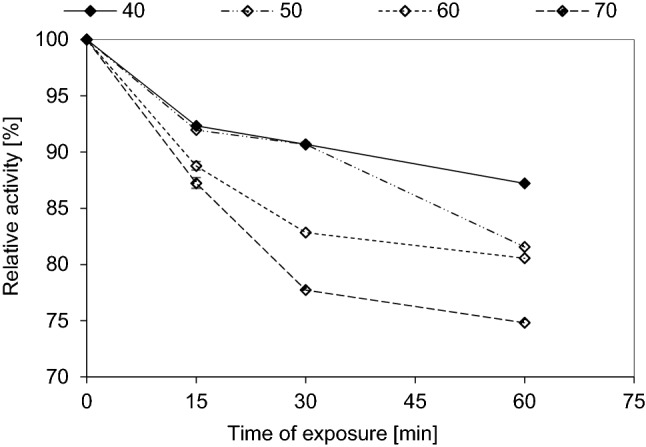
Thermal stability of the purified β-1,3–1,4-glucanase from *Halomonas meridiana* ES021, the enzyme was incubated at different temperatures (40, 50, 60 and 70 ℃) for different periods of time (15, 30, 60 min) in absence of its substrate, then the enzyme assay was performed under optimum reaction condition and the residual enzyme activity was determined. The relative activity was defined as the percentage of activity determined with respect to the maximum β-1,3–1,4-glucanase activity

Thermal stability of β-1,3–1,4-glucanase enzyme was affected by different temperatures and different periods of exposure. β-1,3–1,4-glucanase enzyme when exposed to 40 °C for up to 15 min lost 7.67% of its activity, while it lost 9.32% and 12.79% of its activity after 30 min and 60 min of exposure, respectively. Also, by increasing the temperature to 50 °C, the enzyme lost 8.04%, 9.32% and 18.44% of its activity after 15, 30 and 60 min of exposure, respectively. A further increase in temperature to 60 °C, it lost 11.24%, 17.16% and 19.35% of its activity after 15, 30 and 60 min of exposure, respectively. At treatment temperature of 70 °C, it lost 12.68%, 22.27% and 25.18% of its activity after 15, 30 and 60 min of exposure, respectively (Fig. 8).

#### Effect of different NaCl concentrations

**Fig. 9:**
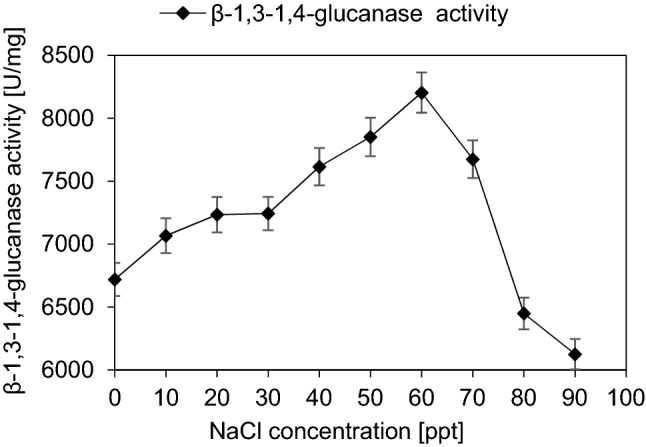
Effect of salinity on the activity of purified β-1,3–1,4-glucanase from *Halomonas meridiana* ES021, the purified enzyme was incubated with different NaCl concentrations ranging from 0 to 90 ppt in absence of its substrate, then the enzyme assay was performed at 40 ℃ and pH 5.0 for 10 min, using a substrate concentration of 0.6 mg/mL reaction mixture. β-1,3–1,4-glucanase is a salt activated enzyme, enzyme activity increased gradually by increasing NaCl concentration and reached the maximum activity at 60 ppt

Β-1,3–1,4-glucanase activity increased gradually by increasing NaCl concentration, the maximum β-1,3–1,4-glucanase activity (8204 ± 160 U/mg) was at NaCl concentration 60 ppt. Further increase in NaCl concentration causes gradual decrease in enzyme activity (Fig. 9).

#### Effect of activators and inhibitors

**Fig. 10:**
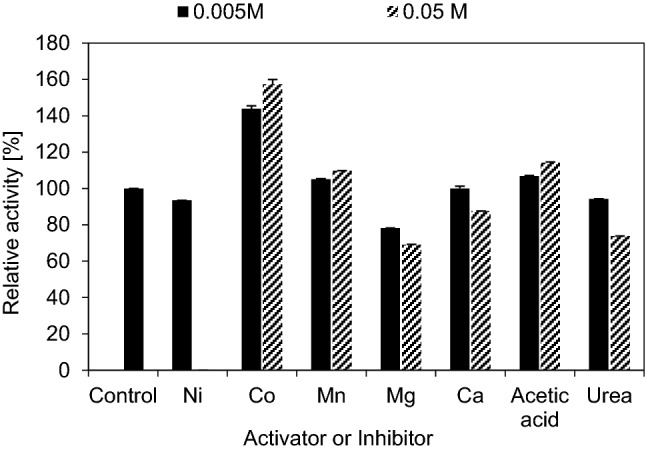
Effect of activators and inhibitors on the activity of purified β-1,3–1,4-glucanase from *Halomonas meridiana* ES021, the purified enzyme was incubated with 0.005 M and 0.05 M concentrations of different metal ions and solvents in absence of its substrate, the enzyme was activated by Co^+2^, Mn^+2^, acetic acid, partially inhibited by Ni^+2^, Mg^+2^, urea and high concentration of Ca^+2^, and completely inhibited by the other tested substances

Different concentrations (0.005 M and 0.05 M) of Co^++^ highly activated β-1,3–1,4-glucanase activity by approximately 43.85% and 57.38%, respectively. 0.005 M and 0.05 M of Mn^++^ also activated β-1,3–1,4-glucanase activity by 5.08% and 9.80%, respectively, while 0.005 M and 0.05 M of acetic acid activated β-1,3–1,4-glucanase activity by 6.83% and 14.11%, respectively. Low concentration of Ni^++^ (0.005 M) slightly inhibited β-1,3–1,4-glucanase activity by approximately 6.50%, while high concentration (0.05 M) completely inhibited the enzyme. Low concentration (0.005 M) of Ca^++^ ions did not affect β-1,3–1,4-glucanase activity while high concentration (0.05 M) inhibited the enzyme by 21.4%. Different concentrations (0.005 M and 0.05 M) of Mg^++^ ions partially inhibited β-1,3–1,4-glucanase activity by approximately by 30.82% and 21.78%, respectively, while different concentrations (0.005 M and 0.05 M) of urea partially inhibited β-1,3–1,4-glucanase activity by approximately 5.70% and 26.09%, respectively. Moreover, β-1,3–1,4-glucanase enzyme was completely inhibited by Cu^++^, Cr^++^ Fe^++^ and SDS (Fig. 10).

### Immobilization of semi-purified β-1,3–1,4-glucanase enzyme produced from *Halomonas meridiana* ES021 cultures

#### Immobilization by physical adsorption and ionic bonding

The immobilized enzyme prepared by adsorption on chitosan showed the highest immobilization activity reached about 429.5 ± 21.0 U/g carrier and the highest immobilization yield reached about 48.71 ± 1.80% among supports used for physical adsorption followed by the immobilized enzyme prepared by adsorption on chitin (230.4 ± 6.0 U/g carrier) with immobilization yield about 26.73 ± 0.09%. The immobilized enzyme prepared by ionic bonding with DEAE-cellulose showed the highest immobilization activity reached about 455.5 ± 21.0 U/g carrier and the highest immobilization yield reached about 51.79 ± 1.77%, even higher than that obtained by physical adsorption on chitosan. However, the immobilized enzyme prepared by ionic bonding with carboxy methyl cellulose (CMC) showed a lower immobilization activity (275.9 ± 10.8 U/g carrier) with immobilization yield about 31.34 ± 0.50% (Table 2).

**Table 2:** Immobilization of semi-purified β-1,3–1,4-glucanase enzyme produced from *Halomonas meridiana* ES021 by physical adsorption and ionic bonding

Carrier	Enzyme added (U/g carrier) (A)	Unbound enzyme (U/g carrier) (B)	Immobilized enzyme (U/g carrier) (I)	Immobilization yield I/(A-B)%
Physical adsorption				
Chitin	1362.5 ± 32.5	500.5 ± 12.9	230.4 ± 6.0	26.73 ± 0.09
Chitosan	1362.5 ± 32.5	480.7 ± 22.0	429.5 ± 21.0	48.71 ± 1.80
Silica gel	1362.5 ± 32.5	366.4 ± 10.0	0.00	0.00
DEAE-Cellulose	1362.5 ± 32.5	318.3 ± 8.0	0.00	0.00
Ionic bonding				
DEAE-Cellulose	1362.5 ± 32.5	627.9 ± 22.0	455.5 ± 21.0	51.79 ± 1.77
CM-Cellulose	1362.5 ± 32.5	626.7 ± 12.0	275.9 ± 10.8	31.34 ± 0.50

#### Immobilization by entrapment in gel material

The immobilized enzyme prepared by entrapment in agarose gel showed the highest immobilization activity reached about 667.08 ± 16.30 U/g carrier and the highest immobilization yield reached about 50.19 ± 0.09%, followed by the immobilized enzyme prepared by entrapment in agar gel (540.42 ± 10.00 U/g carrier) with immobilization yield about 40.66 ± 0.16%. The immobilized enzyme prepared by entrapment in Ca-alginate beads showed the lowest immobilization activity (19.17 ± 0.83 U/g carrier) and the lowest immobilization yield (1.44 ± 0.03%) (Table 3).

**Table 3:** Immobilization of semi-purified β-1,3–1,4-glucanase enzyme produced from *Halomonas meridiana* ES021 by entrapment in gel material

Carrier	Enzyme added (U/g carrier) (A)	Immobilized enzyme (U/g carrier) (I)	Immobilization yield I/A%
Agar	1329.2 ± 30.00	540.42 ± 10.00	40.66 ± 0.16
Agarose	1329.2 ± 30.00	667.08 ± 16.30	50.19 ± 0.09
Ca-Alginate	1329.2 ± 30.00	19.17 ± 0.83	1.44 ± 0.03
Kappa-carrageenan	1329.2 ± 30.00	0.00	0.00

#### Immobilization by covalent bonding

The immobilized enzyme prepared by covalent bonding with 1% glutaraldehyde showed the highest immobilization yield reached about 62.09 ± 0.26% followed by the immobilized enzyme prepared by covalent bonding with 3% glutaraldehyde with immobilization yield about 52.67 ± 0.63%. On the other hand, the immobilized enzyme prepared by covalent bonding with 1% glutaraldehyde showed the lowest immobilization activity (420.4 ± 8.0 U/g carrier) followed by the immobilized enzyme prepared by covalent bonding with 5% glutaraldehyde with immobilization activity about 441.2 ± 11.0 U/g carrier (Table 4).

**Table 4:** Immobilization of semi-purified β-1,3–1,4-glucanase enzyme produced from *Halomonas meridiana* ES021 by covalent bonding

Glutaraldehyde concentrations (%)	Enzyme added (U/g carrier) (A)	Unbound enzyme (U/g carrier) (B)	Immobilized enzyme (U/g carrier) (I)	Immobilization yield I/(A-B)%
1	1329.2 ± 30.0	652.1 ± 20.0	420.4 ± 8.0	62.09 ± 0.26
3	1329.2 ± 30.0	365.8 ± 13.0	507.5 ± 5.0	52.67 ± 0.63
5	1329.2 ± 30.0	445.4 ± 11.0	441.2 ± 11.0	49.92 ± 0.17

### Applications of β-1,3–1,4-glucanase enzyme produced by *Halomonas meridiana* ES021 cultures

#### Enzybiotic activity of purified β-1,3–1,4-glucanase enzyme

**Fig. 11:**
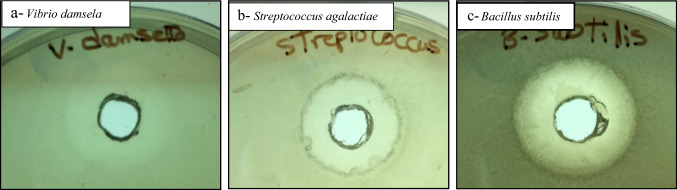
Inhibitory effect of purified β-1,3–1,4-glucanase enzyme from *Halomonas meridiana* ES021 on bacterial growth of a: *Vibrio damsela*; b:* Streptococcus agalactiae*; c:* Bacillus subtilis* in response to 25 µg of enzyme protein

**Fig. 12:**
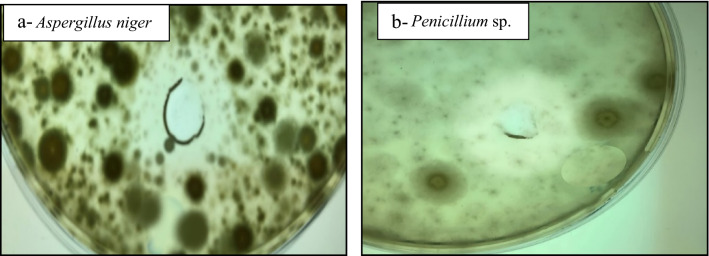
Inhibitory effect of purified β-1,3–1,4-glucanase enzyme from *Halomonas meridiana* ES021 on fungal growth of a: *Aspergillus niger*; b: *Penicillium* sp., in response to 25 µg of enzyme protein

The antibacterial and antifungal effects of purified β-1,3–1,4-glucanase were evaluated. The purified enzyme has antimicrobial effect and it could be used as enzybiotic alternative for treating some bacterial and fungal infections. It has a strong antibacterial effect against *Bacillus subtilis*, *Streptococcus agalactiae* and *Vibrio damsela* strains and weak antibacterial effect on *Escherichia coli*, *Enterococcus faecalis* and *Klebsiella pneumonia*. On the other hand, it has no effect on *Pseudomonas fluorescence*, *Aeromonas hydrophilia*, *Staphylococcus aureus* and *Pseudomonas aeruginosa*. The highest inhibition zone was detected against *Vibrio damsela* followed by *Streptococcus agalactiae* and *Bacillus subtilis*. It also showed high antifungal effect against *Penicillium* sp. followed by *Aspergillus niger.* On the other hand, it has no effect on *Aspergillus oryzae.* Data shown in Table 5 and Fig. 11, 12.

**Table 5:** Enzybiotic activity of purified β-1,3–1,4-glucanase enzyme produced from *Halomonas meridiana* ES021 against bacterial and fungal pathogens

Tested microbial pathogens	ATCC	Inhibition zone diameter (mm)
Bacterial pathogens		
*Vibrio damsela*	–	27
*Streptococcus agalactiae*	6187	21
*Bacillus subtilis*	6633	16
*Escherichia coli*	8739	weak inhibition
*Enterococcus faecalis*	29,212	weak inhibition
*Klebsiella pneumonia*	13,883	weak inhibition
*Pseudomonas fluorescence*	50,090	− ve
*Aeromonas hydrophilia*	914	− ve
*Staphylococcus aureus*	25,923	− ve
*Pseudomonas aeruginosa*	9027	− ve
Fungal pathogens		
*Penicillium sp.*	–	29
*Aspergillus niger*	–	21
*Aspergillus oryzae*	–	− ve

#### Juice clarification by semi-purified β-1,3–1,4-glucanase enzyme

Clarifying of some fruit juices using purified β-1,3–1,4-glucanase was studied. Semi-purified β-1,3–1,4-glucanase was effective in clarification of different juices at different pH values and different time intervals (2 and 4 h). The maximum clarification yield (51.61% and 66.67%) was on mango juice at 40 °C and pH 5.3 for 2 and 4 h, respectively. On the other hand, the lowest clarification yield (20.51%) was on orange juice at 40 °C and pH 4.3 for 2 and 4 h (Table 6).

**Table 6:** Juice clarification by semi-purified β-1,3–1,4-glucanase enzyme produced from *Halomonas meridiana* ES021 cultures

Juice	pH	Incubation period (h)	Control transmission	Sample transmission	Clarification %
Apple	4.5	2	1.08	1.48	37.03
		4	1.18	1.70	44.07
Orange	4.3	2	0.39	0.47	20.51
		4	0.39	0.47	20.51
Guava	4.7	2	0.26	0.35	34.61
		4	0.26	0.35	34.61
Mango	5.3	2	0.31	0.47	51.61
		4	0.30	0.50	66.67
Strawberry	4.2	2	0.20	0.28	40.00
		4	0.20	0.28	40.00

#### Toxicity of purified β-1,3–1,4-glucanase enzyme produced from *Halomonas meridiana* ES021 cultures

The results showed that, the purified β-1,3–1,4-glucanase enzyme showed no toxicity effect on tested *Artemia salina* at all tested concentrations ranging from 20 to 120 µg/mL after 24 and 48 h.

## Discussion

Β-1,3–1,4-glucanase has recently gained a great attention because of its potential applications in food industry (McCarthy et al. 2005; Chaari et al. 2014), feed industry (Beckmann et al. 2006), energy industry (Menon et al. 2011), and its antibacterial effect and cytotoxicity (Jin et al. 2011).

Semi-purified β-1,3–1,4-glucanase was obtained by 75% acetone concentration which gave the highest specific activity, in contrast with Jung et al. (2007); Hong et al. (2009); Tang et al. (2012); Mao et al. (2013); Niu et al. (2016b) and Yang et al. (2014) who used different ammonium sulfate saturations for β-1,3–1,4-glucanase precipitation. Purification of 75% acetone fraction was achieved by anion exchange chromatography with purification fold (3.87-fold) higher than that reported by Hua et al. (2010) (1.29-fold), but it still lower than the purification fold of the recombinant enzymes purified by Yang et al. (2008); Tang et al. (2012); Sun et al. (2012) and Mao et al. (2013). However, Niu et al. (2016b) used cation exchange chromatography for *Bacillus methylotrophicus* S2 β-1,3–1,4-glucanase purification. β-1,3–1,4-glucanase purification was also achieved by other several chromatographic procedures including affinity chromatography (Apiraksakorn et al. 2008; Kim et al. 2013), HPLC using a gel filtration column (Elgharbi et al. 2013) and Ni-IDA column (Zhang et al. 2017). Although the non-*Bacillus* β-1,3–1,4-glucanases are usually larger than the Bacillus enzymes because of additional domains (Planas 2000), *Halomonas meridiana* ES021 β-1,3–1,4-glucanase enzyme has a molecular mass about 72 kDa which was similar to that purified from *Bacillus pumilus* US570 (75 kDa) (Elgharbi et al. 2017). It was smaller than other non-Bacillus β-1,3–1,4-glucanases as reported by *Ruminococcus flavofaciens* (Flint et al. 1993) and *Pseudoalteromonas* (Nakatani et al. 2012) with molecular masses about 90 kDa.

The K_m_ value of the purified enzyme (0.62 mg β-1,3–1,4-glucan/mL) was similar to that reported for fungal β-1,3–1,4-glucanases from *Neocallimastix patriciarum* (0.67 mg/mL) and *Malbranchea cinnamomea* (0.69 mg/mL) (Hung et al. 2012; Yang et al. 2014) and lower than the k_m_ value of β-1,3–1,4-glucanases from *Thermotoga maritima* MSB8 (0.78 mg/mL) (Khan et al. 2007), *Bacillus subtilis* MA139 (0.91 mg/mL) (Pei et al. 2015), *Bacillus pumilus* US570 (2.2 mg/mL) (Elgharbi et al. 2017), and the recombinant β-1,3–1,4-glucanase cloned from *Clostridium Thermocellum* (2.7 mg/mL) (Luo et al. 2014). The V_max_ value (7936 U/mg) was higher than that reported for β-1,3–1,4-glucanase enzyme from truncated *Fibrobacter succinogenes* 7833 U/mg (Chen et al. 2010) and for the recombinant β-1,3–1,4-glucanase enzyme from *Bacillus altitudinis* YC-9 (7692 U/mg) (Mao et al. 2013), and *Bacillus amyloliquefaciens* and *Bacillus tequilensis* CGX5-1 (7500 U/mg) (Sun et al. 2012; Wang et al. 2014).

The optimum temperature for reaction was 40 °C, showed specific activity about 5131 ± 147 U/mg, which was higher than that reported for recombinant β-1,3–1,4-glucanase purified from *Bacillus licheniformis* EGW039 (2,479 U/mg) (Teng et al. 2006). This temperature was similar to that reported for recombinant β-1,3–1,4-glucanase purified from *Paenibacillus* sp. X4 and *Bacillus* sp. SJ-10 cultures (Baek et al. 2017; Tak et al. 2019), and for both wild type and mutant β-1,3–1,4-glucanase purified from *Bacillus subtilis* MA139 (Pei et al. 2015). Also, it was higher than that reported for recombinant β-1,3–1,4-glucanase purified from *Saccharophagus degradans* (30 °C) (Lafond et al. 2016), and *Bacillus velezensis* ZJ20 (35 °C) (Xu et al. 2016). The purified β-1,3–1,4-glucanase enzyme retained more than 80% of its activity in a temperature range from 30 to 70 °C similarly to Kim et al. (2013), similar range was also reported for recombinant β-1,3–1,4-glucanase purified from *Bacillus terquilensis* CGX 5–2 while its wildtype could only maintain high activity in a temperature range from 35 to 55 °C (Niu et al. 2017).

The optimum pH value of the purified β-1,3–1,4-glucanase enzyme was pH 5.0, this value is the same as the optimal pH values of β-1,3–1,4-glucanases from *Bacillus subtilis* (Fu et al. 2008), *Bacillus altitudinis* (Mao et al. 2016), *Paenibacillus* sp. (Baek et al. 2017), *Aspergillus fumigatus* (Bernardi et al. 2018) and *Aspergillus awamori* (Liu et al. 2020), and higher than those of β-1,3–1,4-glucanases from *Trichoderma koningii ZJU-T* (pH 2.0) (Wang et al. 2007), *Penicillium occitanis* Pol6 (pH 3.0) (Chaari et al. 2014) and *Laetiporus sulphureus* var. *miniatus* (pH 4.0) (Hong et al. 2009). The purified β-1,3–1,4-glucanase enzyme retained more than 90% of its activity in a pH range from 4.0 to 7.0, similarly to Chaari and Chaabouni (2019) while in Chaari et al. (2012) research, it retained only 80% of its activity in the same pH range.

The purified β-1,3–1,4-glucanase enzyme was stable to heat treatment in absence of its substrate. It retained about 90% of its activity when exposed to 40 and 50 °C for up to 45 min, while it retained only 81.56% of its activity when exposed to 50 °C for 60 min, this thermal stability was comparable to that of β-1,3–1,4-glucanases purified from *Bacillus licheniformis* (Chaari et al. 2012), and *Bacillus *sp. SJ-10 (Jang et al. 2021). By increasing the temperature to 60 °C, the enzyme activity decreased by only about 20% of the original activity after 60 min of exposure, so it considered to be more thermostable than the β-1,3–1,4-glucanase produced from *Paenibacillus* sp. which lost about 35% of its activity at the same conditions (Baek et al. 2017). *Halomonas meridiana* ES021 β-1,3–1,4-glucanase enzyme retained about 75% of its activity when exposed to 70 °C for up to 60 min, while the recombinant β-1,3–1,4-glucanase purified from *Bacillus altitudinis* could retained 75% of its activity for only 10 min (Mao et al. 2013), and that purified from *Bacillus tequilensis* retained only about 60% of activity for 60 min at the same temperature (Niu et al. 2016a).

*Halomonas meridiana* ES021 β-1,3–1,4-glucanase is a halotolerant enzyme, it retained more than 90% of its activity after exposure to 90 ppt NaCl. Also, gradual increase in enzyme activity by increasing NaCl concentration indicated that it is a salt activated enzyme similarly to the recombinant enzyme cloned from *Paenibacillus* sp. S09 (Cheng et al. 2014). At 60 ppt, the enzyme activity increased about 122% and this result was higher than that of fungal β-1,3–1,4-glucanase purified from *Aspergillus fumigatus* which increased only 113.2% when treated with 5 mM NaCl.

Co^++^ was the best activating metal ion with a relative activity about 157.38% which was higher than those reported for bacterial β-1,3–1,4-glucanases from *Bacillus altitudinis* YC-9 (124.78%) (Mao et al. 2013), *Bacillus licheniformis* GZ-2 (116.6%) (Gao 2016), and fungal β-1,3–1,4-glucanases from *Paecilomyces thermophila* (117%) (Yang et al. 2008), *Laetiporus sulphureus* var. *miniatus* (109%) (Hong et al. 2009), *albranchea cinnamomea* (147.2%) (Yang et al. 2014), while it is slightly lower the relative activities of fungal β-1,3–1,4-glucanases from *Aspergillus fumigatus* (161.1%) (Bernardi et al. 2018), and *Thermoascus aurantiacus* (159.8%) (Yan et al. 2018). Recombinant β-1,3–1,4-glucanases from *Bacillus licheniformis*,* Bacillus subtilis* and *Paenibacillus* sp. were also activated by Co^++^ ions (Teng et al. 2007; Jung et al. 2010; Chang et al. 2011). In contrast with these results some investigators reported an inhibitory effect of Co^++^ ions on β-1,3–1,4-glucanase (Chaari et al. 2012; Elgharbi et al. 2013; Chaari et al. 2014; Wang et al. 2014; Elgharbi et al. 2017; Ali et al. 2019). The enzyme was also slightly activated in presence of Mn^++^, this result agreed with the data reported by (Teng et al. 2007; Kim et al. 2014; Yan et al. 2018), while disagreed with (Wang et al. 2007; Apiraksakorn et al. 2008; Hong et al. 2009; Luo et al. 2014; Pei et al. 2015; Gao 2016) who reported partial inhibition effect of Mn^++^ ions while complete inhibition of the enzyme was reported by (Yang et al. 2008; Elgharbi et al. 2013; Chaari et al. 2014; Kim et al. 2014; Elgharbi et al. 2017).

On the other hand, *Halomonas meridiana* ES021 β-1,3–1,4-glucanase was partially inhibited by low concentration of Ni^++^, while the high concentration completely inhibited it. The inhibitory effect of Ni^++^ was also reported by Yang et al. (2008) in contrast with Hong et al. (2009), Yang et al. (2014), Gao (2016) and Bernardi et al. (2018) as they reported activation of β-1,3–1,4-glucanase with Ni^++^ ions. Mg^++^ ions inhibited β-1,3–1,4-glucanase activity, similar effect was reported by Celestino et al. (2006), Wang et al. (2007), Apiraksakorn et al. (2008), Yang et al. (2008), Hong et al. (2009), Chaari et al. (2014), Yang et al. (2014) and Elgharbi et al. (2017). This result was dissimilar to the activating effect of Mg^++^ ions recorded by Jung et al. (2010), Cheng et al. (2014), Zhang et al. (2017) and Schröder et al. (2018). Partial inhibition of the enzyme occurred by high concentration of Ca^++^, while no change in activity observed with low level, similarly to these data slight inhibition with Ca^++^ ions was reported by Apiraksakorn et al. (2008), Elgharbi et al. (2013) and Wang et al. (2014) and dissimilarly Ca^++^ ions made activation for other β-1,3–1,4-glucanases (Gao 2016; Elgharbi et al. 2017; Bernardi et al. 2018). Complete inhibition of *Halomonas meridiana* ES021 β-1,3–1,4-glucanase was also observed with Cu^++^ ions, similarly to Yang et al. (2008), Elgharbi et al. (2013) and Pei et al. (2015), this was disagreed with the activating effect reported by Celestino et al. (2006), Wang et al. (2007), Yang et al. (2014) and Niu et al. (2016b) for the same metal ion. Urea slightly inhibited the enzyme activity, similar result was reported by Wang et al. (2007). Moreover, Fe^++^ ions completely inhibited the enzyme activity, similarly to Elgharbi et al. (2013) and Ali et al. (2019), other investigators reported activation of β-1,3–1,4-glucanase by Fe^++^ ions (Teng et al. 2007; Mao et al. 2013; Chaari et al. 2014; Kim et al. 2014; Bernardi et al. 2018). Furthermore, complete inhibition occurred by sodium dodecyl sulfate (SDS), this result agreed with Bernardi et al. (2018). However, Wang et al. (2014), Yang et al. (2014) and Niu et al. (2016b) reported that SDS has activation effect on β-1,3–1,4-glucanase. Finally, Cr^++^ ions and acetic acid caused complete inhibition of the enzyme activity, but no research articles studied their effect on β-1,3–1,4-glucanase till now.

Results obtained by enzyme immobilization on porous silica gel and DEAE-cellulose by adsorption indicate that weak adsorption occurred on both carriers, so the enzyme was eluted by washing process and no immobilization yield detected, while immobilization by adsorption on chitosan and ionic binding on DEAE-Cellulose showed relatively high immobilization yields. Good immobilization yields were also achieved by entrapment in agar and agarose gel materials. This research is the first report for β-1,3–1,4-glucanases immobilization using these techniques. Covalent bonding on chitosan using 1% glutaraldehyde as cross-linking reagent was the best immobilization technique with the highest immobilization yield, similar technique was used by Cho et al. (2018) to covalently immobilize *Bacillus* sp. β-1,3–1,4-glucanase enzyme on porous silica using glutaraldehyde as crosslinking reagent. A recent research covalently immobilized β-1,3–1,4-glucanase from *Penicillium occitanis* successfully on chitosan–clay composite beads using glutaraldehyde as cross-linking reagent (Chaari et al. 2015).

Purified *Halomonas meridiana* ES021 β-1,3–1,4-glucanase enzyme exhibited an enzybiotic activity against some newly studied pathogens, this activity was varying between bacterial and fungal pathogens. β-1,3–1,4-glucanase showed an antibacterial effect against *Bacillus subtilis*, *Escherichia coli* and *Enterococcus faecalis* similarly to Jin et al. (2011), while its activity against *Streptococcus agalactiae*, *Vibrio damsela* and *Klebsiella pneumonia* was first approved in this study. On fungi level, antifungal effect was reported against *Penicillium* sp. (human pathogenic), Yuan et al. (2020) also reported the antifungal effect of β-1,3–1,4-glucanase against *Canidia albicans* (human pathogenic yeast). The enzyme also showed a weak antifungal effect against the phytopathogenic *Aspergillus niger*, the antifungal activity of the enzyme against phytopathogenic fungi was previously reported against *Curvularia affinis* and* Colletotrichum gloeosporioides* (Dewi et al. 2016), *Cryphonectria parasitica*,* Cylindrocladium quinqueseptatumI, Helicobasidium purpureum* (Xu et al. 2016), and *Alternaria alternata* (Zalila‐Kolsi et al. 2018). Juice clarification by semi-purified β-1,3–1,4-glucanase enzyme showed variation in clarification yields of different juices at their different pH values. The obtained results observed that the purified β-1,3–1,4-glucanase enzyme could be used to reduce the cost of juice processes. β-1,3–1,4-glucanase enzyme was first used as a juice clarifying agent in this study, while juice clarification was processed by other type of β-glucanases as cellulase (β-1,4-glucanase) (Pradhan et al. 2021), and by combination of cellulase with pectinase (Al-Hooti et al. 2002; Abbès et al. 2011). In order to ensure the enzyme safety, toxicity of purified β-1,3–1,4-glucanase enzyme was tested on *Artemia salina* showing no toxicity at different tested concentrations. This result indicated that β-1,3–1,4-glucanase could be a safe supplement to be used in food and drug industry and other biotechnological applications, as well as it could be applied as a safe prebiotic substance to enhance the immune system of aquacultures of different species also, prebiotics are known as a substrate for probiotic commensal bacteria and can induce immune response (Pujari and Banerjee 2021).

## Conclusion

A novel halotolerant β-1,3–1,4-glucanase was successfully purified from the cultures of marine *Halomonas meridiana*. This study demonstrated the enzybiotic activity of β-1,3–1,4-glucanase against newly studied pathogenic bacterial and fungal strains, and its potential to be used as a natural and safe clarifying agent in juice industry. The thermal stability and the high immobilization efficiency make this enzyme of potential interest in a number of industrial applications. Also, β-1,3–1,4-glucanase has no toxicity on *Artemia salina*, so it could be safely applied in various industries especially in aquaculture.

## Data Availability

The datasets generated during the current study are available in Digital Library of Alexandria University.
